# No Emergence of Colistin Resistance in the Respiratory Tract of Lung Transplant Patients Treated With Inhaled Colistin

**DOI:** 10.3389/ti.2024.13545

**Published:** 2025-01-23

**Authors:** Nathalie Grall, Maksud Assadi, Marina Esposito-Farese, Brice Lortat-Jacob, Sébastien Tanaka, Enora Atchade, Jonathan Messika, Vincent Bunel, Hervé Mal, Pierre Mordant, Yves Castier, Bastien Garnier, Signara Gueye, Marie Petitjean, Erick Denamur, Laurence Armand-Lefevre, Charles Burdet, Philippe Montravers, Alexy Tran-Dinh

**Affiliations:** ^1^ Université Paris Cité, AP-HP, Hôpital Bichat Claude Bernard, Service de Bactériologie, Paris, France; ^2^ INSERM UMR 1137 IAME, Université Paris Cité, Paris, France; ^3^ Université Paris Cité, AP-HP, Hôpital Bichat Claude Bernard, Département d’Anesthésie-Réanimation, Paris, France; ^4^ Université Paris Cité, AP-HP, Hôpital Bichat, Département d’Epidémiologie et Recherche Clinique, Paris, France; ^5^ Réunion Island University, INSERM U1188 Diabetes Atherothrombosis Réunion Indian OCean (DéTROI), CYROI Plateform, Saint-Denis de La Réunion, France; ^6^ Université Paris Cité, AP-HP, Hôpital Bichat Claude Bernard, Pneumologie B et Transplantation Pulmonaire, Paris, France; ^7^ INSERM UMR 1152 PHERE, Université Paris Cité, Paris, France; ^8^ Paris Transplant Group, Paris, France; ^9^ Université Paris Cité, AP-HP, Hôpital Bichat Claude Bernard, Service de Chirurgie Vasculaire, Thoracique et Transplantation Pulmonaire, Paris, France; ^10^ INSERM UMR 1148 LVTS, Université Paris Cité, Paris, France

**Keywords:** colistin, resistance, lung transplantation, prophylaxis, pneumonia

## Abstract

Secondary prophylaxis using inhaled colistin (IC) was implemented to prevent recurrences of Pseudomonas aeruginosa or extended-spectrum β-lactamase-producing Enterobacterales (ESBL-PE) pneumonia during the postoperative intensive care unit (ICU) stay after lung transplantation (LT). We evaluated the risk of emergence of colistin resistance in the respiratory tract during secondary IC prophylaxis. We conducted a prospective, single-centre, observational study of all adult patients who underwent LT between 1 July 2018 and 30 June 2019. IC was started and continued for at least 90 days for *P. aeruginosa* or ESBL-PE pneumonia. During the 90 days following LT, all respiratory samples were routinely tested for the presence of GNB of reduced susceptibility to colistin. Twenty-seven (38.6%) of the 70 included patients received IC. Among the 867 respiratory samples tested, IC did not promote the emergence of bacterial species with natural or acquired resistance to colistin (incidence-rate ratio of 0.21 [0.03–1.58], *p* = 0.13 and 1.68 [0.55–5.12], *p* = 0.37, respectively). Our study suggests no association between the use of IC and an increased risk of colistin resistance in the respiratory tract within 90 days of LT.

## Graphical Abstract

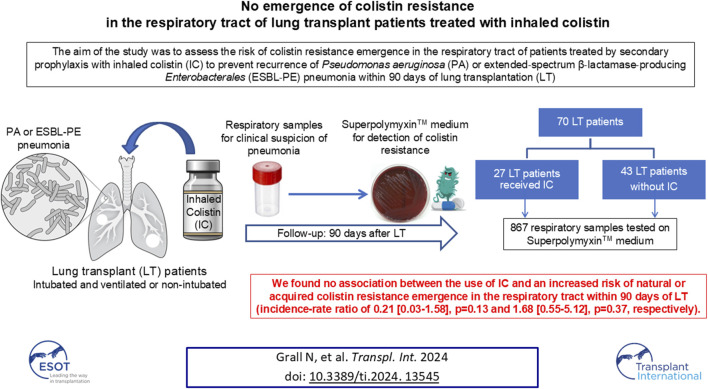

## Introduction

Lung transplantation (LT) is a last-resort therapy for patients with end-stage lung diseases. Nearly three-fourths of patients experience at least one episode of pneumonia within 1 year of LT, especially within the first month, and this complication is an independent risk factor for 1-year mortality [1]. Gram-negative bacilli (GNB), led by *Pseudomonas aeruginosa*, are the most common infectious agents causing pneumonia in lung transplant patients [1]. In addition, *P. aeruginosa* airway colonization increases the risk of chronic lung allograft dysfunction [2, 3]. Since January 2018, we implemented secondary prophylaxis using inhaled colistin (IC) at our institution to prevent recurrences of *P. aeruginosa* or extended-spectrum β-lactamase-producing *Enterobacterales* (ESBL-PE) pneumonia during the postoperative intensive care unit (ICU) stay after LT. In a before-and-after retrospective cohort analysis of 271 LT patients, including 125 recipients in the observation period before the use of secondary prophylaxis with IC, and 146 recipients in the intervention period with the use of secondary prophylaxis with IC, we showed that the use of IC as secondary prophylaxis decreased the proportion of patients who experienced at least one recurrence of *P. aeruginosa* or ESBL-PE pneumonia (7.2% during the observation period versus 0.7% during the intervention period, *p* = 0.007) [4]. Colistin belongs to the polymyxin family and has significant antibacterial activity against GNB by targeting and disrupting lipopolysaccharides in the outer cell membrane [5]. Because colistin is often used as a last line antibiotic in multidrug-resistant GNB infections [6], the risk of emergence of acquired resistance to colistin is of concern, especially since the identification of the first plasmid gene for colistin resistance, *mcr* [7]. The latest report from the French National Reference Center for Resistance to Antibiotics reported a 2%–4% prevalence of colistin-resistant *P. aeruginosa* strains in 2019 [8]. The present study evaluated the risk of emergence of colistin resistance in the respiratory tract during secondary IC prophylaxis introduced for *P. aeruginosa* or ESBL-PE pneumonia in the ICU after LT.

## Materials and Methods

### Study Design

We performed a prospective, single-centre, observational study (Bichat-Claude Bernard Hospital, Paris, France). The studies involving humans were approved by Ethical authorizations were obtained from the National Ethics Committee for the Protection of Persons Nord-Ouest (N°034/2018). The studies were conducted in accordance with the local legislation and institutional requirements. The participants provided their written informed consent to participate in this study. No animal studies are presented in this manuscript. No potentially identifiable images or data are presented in this study. The raw data supporting the conclusions of this article will be made available by the authors, without undue reservation.

All adult patients who underwent LT between 1 July 2018 and 30 June 2019 were included.

The included patients were followed up for 90 days. Surgical transplantation procedures and perioperative care, including postoperative and immunosuppressive management, were standardised for all patients according to our local protocol as previously described [9]. Cefazolin (or the antibiotic that was administered to the donor at harvest) was used as the standard antibiotic prophylaxis and was adapted to microbiological cultures obtained from bronchoalveolar lavage (BAL), which was systematically performed just after surgery. Antibiotic prophylaxis was stopped after 48 h in patients with negative cultures of postoperative BAL, as recommended [10]. IC was started [3 Million International Units (MIU) twice daily] in combination with intravenous antibiotic therapy in cases of *P. aeruginosa* or ESBL-PE pneumonia during the postoperative ICU stay, which were diagnosed from the recommendations for the standardisation of definitions of infections in cardiothoracic transplant recipients [11], as previously described elsewhere [4]. IC was used as secondary prophylaxis on the assumption that it could prevent recurrence of *P. aeruginosa* or ESBL-PE pneumonia, and intravenous antibiotic therapy was used to curatively treat the *P. aeruginosa* or ESBL-PE pneumonia episode according to the recommendations [12–14]. IC was continued for at least 90 days, regardless of whether the patient was still on mechanical ventilation. After 90 days, continuation of this treatment was left to the discretion of the physician in charge. The duration of intravenous antibiotic therapy was generally 7 days, but could be longer depending on the doctor’s decision.

### Data Collection

We recorded patient characteristics at baseline (age, sex, body mass index, and aetiology of pulmonary disease), type of LT (i.e., single or double LT), rate of pneumonia, duration of mechanical ventilation, length of ICU stay, tracheostomy, time from LT and initiation of IC, duration of IC treatment within 3 months of LT, IC-related side effects, exposure to antibiotics within 3 months, specific lung graft complications (acute cellular rejection confirmed by histopathological evidence after transbronchial lung biopsies performed only in cases of suspicion and not systematically [15]; definite, probable or possible antibody-mediated rejection according to Levine et al [16]); and airway complications that were severe bronchial stenosis requiring balloon dilation or insertion of endobronchial stent and bronchial anastomosis dehiscence [17], ICU and mortality rates at 28 days and 90 days.

### Microbiological Analysis

During the 90 days following LT, all respiratory samples [plugged telescoping catheter (PTC), BAL, bronchial aspirate (BA), sputum] were only taken during usual care (i.e., when pneumonia was suspected) during the postoperative ICU stay, conventional pulmonology hospitalisation and day hospital and systematically plated on a selective medium (SuperPolymyxinTM, Ellitech, Puteaux, France). There was no systematic respiratory sampling protocol to detect tracheobronchial colonization. Samples were incubated at 37°C for 48 h, in addition to the standard cultures. This selective medium allows for the detection of GNBs with reduced susceptibility to polymyxins, including colistin, regardless of the mechanism or level of resistance. All distinct colonies were studied. Identification was performed using mass spectrometry (Maldi Biotyper^®^, Bruker Daltonics, Bremen, Germany). The susceptibility to antibiotics was determined using the disk diffusion method according to the recommendations of EUCAST.^1^ ESBL production was confirmed using the double-disk synergy test [18]. We distinguished GNB with natural resistance to colistin (*Proteus* spp., *Providencia* spp., *Serratia* spp., *Morganella* spp., *Hafnia alvei*) from GNB with acquired resistance to colistin. The minimum inhibitory concentration (MIC) of colistin was determined using microdilution (Umic, Biocentric, Bandol, France) for all strains naturally susceptible to colistin. Resistance to colistin was defined as an MIC >2 mg/L (EUCAST).

### Whole Genome Sequencing and Analysis

To determine the colistin resistance mechanism, whole genome sequencing (WGS) of *Enterobacterales* and *P. aeruginosa* isolates with acquired resistance to colistin was performed on each isolate of the same species with identical antibiotic susceptibility per patient. WGS was performed on a MiniSeq system (Illumina, San Diego, United States) with paired-end reads and read lengths of 150 bases. Libraries were prepared using the Nextera DNA Sample Preparation Kit from Illumina. Reads from Illumina sequencing were used for whole genome analyses. Read quality was assessed using FastQC v0.11.8. and Trim Galore v0.4.5 was used for quality and adapter trimming. Trim Galore was set up to trim basecalls with a Phred quality score inferior to 30, and reads less than 50 bases long were withdrawn. MetaPhlAn2 v2.6.0 [19] was used to verify the identifications of isolates and identify putative cross-contaminations. Reads were assembled using SPAdes v3.11.1 [20]. The quality of the assemblies was examined using QUAST v5.0.2 [21]. Gene annotation was performed using Prokka v1.13.3 [22].

The sequence type (ST) of the isolates was determined using CGE MLST software [23]. Diamond [24] was used to identify all of the antibiotic resistance genes by aligning all genomes against the AMRFinder database (version 2019-04-29). To obtain a reference genome, *Enterobacter cloacae* and *Klebsiella aerogenes* strains were downloaded from the Genome Taxonomy Database (GTDB) [25], and the closeness of the strain was tested using Mash [26]. For *Escherichia coli*, strains from the same phylogroup were downloaded from the EnteroBase database [27]. We used the closest strain to avoid SNPs linked to evolution. For *P. aeruginosa*, PAO1 genes were downloaded from NCBI. Using CD-HIT v4.7 [28], interesting genes (*pho*P, *pho*Q, *pmr*A, *pmr*B and *mgr*B/*yob*G for *Enterobacterales* and *pho*P, *pho*Q, *pmr*A, *pmr*B, *par*R, *par*S, *col*R, *col*S, *cpr*R and *cpr*S for *P. aeruginosa*) were searched in the genomes of our strains. Polymorphisms in these genes were determined using ClustalOmega v1.2.4 [29] for alignment against the reference genome downloaded from NCBI and a Python script for specific SNP detection. The impact of mutations detected was assessed using SIFT [30], PROVEAN [31] and Polyphen-3 [32]. We considered a deleterious effect for the mutation if two of these software packages predicted a deleterious effect.

### Statistical Methods

Data are presented as medians and interquartile ranges for continuous variables and as frequencies and percentages for categorical variables.

We compared the baseline characteristics of patients and outcomes according to their exposure to inhaled colistin using Fisher’s exact or Wilcoxon tests, as appropriate.

The incidence rate of colistin-resistant GNB emergence in the respiratory tract in the 90 days following LT was estimated by pooling the GNB with acquired or natural resistance to colistin. To control for the immortal-time bias induced by direct comparison of exposed and unexposed patients to IC treatment, we applied the statistical methodology described and developed by Suissa [33, 34]. The immortal-time bias is the bias induced by the period before exposure to IC treatment in patients who will be exposed at a given time. During this unexposed period, a bias occurs because no events may be observed under exposure. According to this approach, comparisons are made between exposed and unexposed person-times, unlike subjects. Following the methods described in a previous study [33], we defined the observation period from LT to 90 days for surviving patients and death for the other patients. Exposure and non-exposure times to IC were identified for each patient, and the emergence of resistance to colistin, if applicable. Incidence rates under exposed and unexposed periods, their ratios and 95% confidence intervals (CI) were estimated using Poisson log-linear regressions.

All tests were 2-sieded, with a type-I error of 0.05. Analyses were performed using R software, version 4.0.5 (Copyright (C) 2021 The R Foundation for Statistical Computing).

## Results

### Patient Demographics and Outcomes After Lung Transplantation

Seventy patients underwent LT during the study period and were included in the present study. No patient was lost to follow-up. The baseline characteristics of the patients are presented in Table 1. Patients were primarily transplanted for chronic obstructive pulmonary disease (COPD) (31.4%) and interstitial lung diseases (ILD) (57.1%), with a median age of 59 [52–63] years and a male/female ratio of 1.80. Three patients (two patients who did not receive IC and one patient who received IC) had been colonized by *P. aeruginosa* prior to lung transplantation. No patient had a history of IC treatment prior to lung transplantation.

**TABLE 1:** Baseline characteristics of patients, early complications and mortality during the 3 months after lung transplantation.

	Overall (n = 70)	Patients with inhaled colistin (n = 27)	Patients without inhaled colistin (n = 43)	*p*-value
Age, years	59 [52: 63]	57 [50: 60.5]	60 [54: 64]	0.83
Female	25 (35.7)	12 (44.4)	13 (30.2)	0.30
LT aetiology				1.00
COPD/Emphysema	22 (31.4)	8 (29.6)	14 (32.6)	
Interstitial lung diseases	40 (57.1)	16 (59.3)	24 (55.8)	
Others	8 (11.4)	3 (11.1)	5 (11.6)	
Type of LT				0.61
Single LT	27 (38.6)	9 (33.3)	18 (41.9)	
Double LT	43 (61.4)	18 (66.7)	25 (58.1)	
BMI, kg/m^2^	25 [22: 28]	26 [23: 29]	24 [21: 26]	0.036
Respiratory samples	11 [9: 16]	13 [11: 19]	11 [8: 14]	0.009
Tracheotomy	24 (34.3)	16 (59.3)	8 (18.6)	0.0007
Mechanical ventilation, days	3 [1: 25]	25 [4: 45]	2 [1: 5]	0.0004
Gram-negative pneumonia	51 (72.9)	27 (100)	24 (55.8)	0.00001
Gram-positive pneumonia	12 (17.1)	5 (18.5)	7 (16.3)	1
ICU length of stay, days	20 [12: 42]	41 [24: 68]	14 [11: 23]	0.0002
Time to IC initiation, days	NA	16 [10: 32]	NA	
Duration of IC exposure, days	NA	74 [60: 80]	NA	
Severe bronchial stenosis	17 (24.3)	8 (29.6)	9 (20.9)	0.57
Bronchial anastomosis dehiscence	6 (8.6)	2 (7.4)	4 (9.3)	1
Acute cellular rejection	19 (27.1)	11 (40.7)	8 (18.6)	0.06
Antibody-mediated rejection	38 (54.3)	17 (63)	21 (48.8)	0.33
ICU mortality	11 (15.7)	4 (14.8)	7 (16.3)	1
D28 mortality	7 (10)	1 (3.7)	4 (9.3)	0.64
D90 mortality	10 (14.3)	3 (11.1)	7 (16.3)	0.73

Categorical and continuous measures are represented as numbers (%) and medians, respectively [Q1: Q3]. LT, lung transplantation; COPD, chronic obstructive pulmonary disease; BMI, body mass index; ECMO, extracorporeal membrane oxygenation; ICU, intensive care unit; NA, not applicable.

**FIGURE 1:**
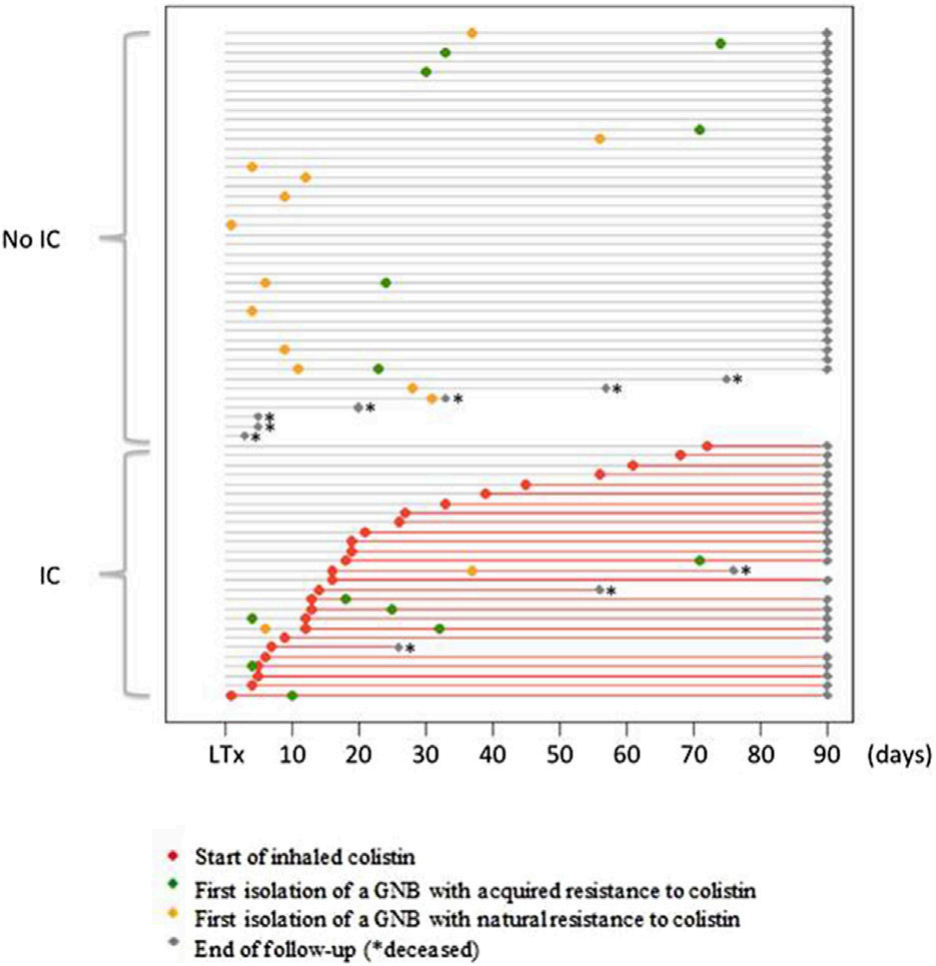
Initiation of colistin treatment and dynamics of colonization by colistin-resistant GNB during the 90 days following lung transplantation. Each horizontal grey line represents the course of one patient, from lung transplantation (LT) to 3 months punctuated by a grey dot or before in case of death (*). The red lines represent the period of inhaled colistin (IC) treatment after initiation (red dot). The time of the first isolation of a Gram-negative bacilli (GNB) with acquired resistance to colistin is represented by a green dot. The time of the first isolation of a Gram-negative bacilli (GNB) with natural resistance to colistin is represented by a yellow dot. Grey dots indicate the time of the end of follow-up, and * indicates the time of death.

Twenty-seven (38.6%) patients received IC (Figure 1). The median time between LT and the initiation of IC was 16 [10–32] days, with a median duration of exposure of 74 [60–80] days. Patients receiving IC experienced important morbidity during the postoperative course in the ICU with a longer duration of mechanical ventilation (25 [4–45] vs. 2 [1–5] days, *p* = 0.0004), more tracheostomies (59.3% vs. 18.6%, *p* = 0.0007), and longer ICU length of stay (41 [24–68] vs. 14 [11–23] days, *p* = 0.0002) than patients who did not receive IC. The rate of GNB pneumonia was higher in patients who received IC than in those who did not (100% vs. 55.8%, *p* = 0.00001), whereas the rate of Gram-positive cocci pneumonia was similar (18.5% vs. 16.3%, *p* = 1). Patients who received IC had similar rates of acute cellular rejection (40.7% vs. 18.6%, *p* = 0.06), antibody-mediated rejection (63% vs. 48.8%, *p* = 0.33), severe bronchial stenosis (29.6% vs. 20.9%, *p* = 0.57), and bronchial anastomosis dehiscence (7.4% vs. 9.3%, *p* = 1) versus patients who did not receive IC. The mortality rates at Day 28 and Day 90 were not significantly different between the patients with and without IC (3.7% vs. 9.3%, *p* = 0.64 and 11.1% vs. 16.3%, *p* = 0.73) (Table 1).

No significant IC-related adverse events were observed, particularly no bronchospasm or acute kidney injury.

### Phenotypic Analysis of the Respiratory Samples

A total of 867 respiratory samples were screened for the presence of GNB resistant to colistin, with 393 (45.3%) BAL, 38 (4.4%) PTC, 420 (48.4%) BA and 16 (1.9%) sputum samples.

The median number of samples collected per patient was 11 [9–16]. Patients receiving IC had more samples than patients without IC (13 [10.5–18.5] vs. 11 [8–14], *p* = 0.009).

#### Emergence of GNB With Natural Resistance to Colistin

A naturally colistin-resistant GNB was isolated in 54 (6.2%) samples (13 BAL, 39 BA and 2 sputum) from 14 (20%) patients. Among these 14 patients, 12 patients never received IC, one patient had his only positive sample before the introduction of IC, and one patient had his only positive sample during IC treatment (Figure 1). The species isolated were *Morganella morganii* (n = 18), *Serratia marcescens* (n = 17), *Proteus mirabilis* (n = 12) and *Hafnia alvei* (n = 7). Incidence rate ratio between exposed and unexposed patients to colistin was 0.21 [0.03–1.58] (*p* = 0.13) (Table 2).

**TABLE 2:** Incidence rates of acquired and natural colistin resistance among the 70 patients included.

Event	First colistin resistant GNB isolation during inhaled colistin treatment	Number of events	Person-days	Incidence rates	IRR [95% CI]	*p*-value
Acquired resistance	Yes	5	1,681	0.0030	1.68 [0.55–5.12]	0.37
	No	8	4,507	0.0020		
Natural resistance	Yes	1	1,681	0.0006	0.21 [0.03–1.58]	0.13
	No	13	4,507	0.0029		

GNB, Gram-negative bacilli; IRR, incidence-rate ratio; CI, confidence interval.

#### Emergence of GNB With Acquired Resistance to Colistin

A GNB with acquired resistance to colistin was isolated in 28/867 (3.2%) samples (14 BAL, 11 BA and 3 sputum) from 13/70 (18.6%) patients. Among these 13 patients, 6 patients never received IC, 2 patients had their first positive sample before the introduction of IC, and 5 patients had their first positive sample during IC treatment. The time between IC introduction and the first isolation of a GNB with acquired colistin resistance ranged from 5 to 53 days. The species isolated were *E. cloacae* (n = 7), *E. coli* (n = 1), *K. aerogenes* (n = 1), *P. aeruginosa* (n = 13) and *Stenotrophomonas maltophilia* (n = 6).

Among the 13 patients with GNB with acquired colistin resistance, 7 patients (3 with IC and 4 without IC) had only one positive sample, and 4 patients (3 with IC and 1 without IC) had 2 positive samples. One patient who never received IC was colonized by an *E. cloacae* strain that was resistant to colistin, with 7 positive samples over a period of 53 days. The last patient had 6 positive samples during IC treatment. A colistin-resistant *K. aerogenes* strain was isolated in the first sample 12 days after IC introduction, and a colistin-resistant *P. aeruginosa* strain was isolated in the 5 other samples between 38 and 77 days after IC introduction.

IC did not promote the emergence of acquired colistin resistance in the respiratory samples, with an incidence-rate ratio of 1.68 [0.55–5.12] (*p* = 0.37) (Table 2).

### Characteristics of Strains With Acquired Colistin Resistance

The MIC to colistin of the 28 GNB isolates with acquired resistance was between 4 and 16 mg/L.

WGS was performed on one isolate of the same species with identical antibiotic susceptibility per patient for *Enterobacterales* and *P. aeruginosa* strains. The results are described in Supplementary Table S1. The 7 isolates of *E. cloacae* isolated from the same patient were ESBL-producing and carried *bla*_CTX-M-15_. No known *mcr* genes (*mcr*-1 to *mcr*-10) were detected. A missense mutation in the *pmr*A gene coding for a two-component system (PmrAB) associated with colistin resistance was identified in the *E. coli* and *K. aerogenes* strains. For both strains, the mutation was localized in G53, which is an amino acid hot spot in PmrA [35]. The mutation was G53C for *E. coli* and G53R for *K. aerogenes*. Both mutations were predicted to impact the protein and were described previously [35]. No mutation was found in the pmrB, phoP, phoQ or mgrB genes. No mutation was found in *pmr*A, pmrB, phoP, phoQ or mgrB genes in the *E. cloacae* strain. This strain belongs to cluster VIII, which is not known to have a heteroresistance phenotype to colistin [36]. Among the eight sequenced *P. aeruginosa* strains, a missense mutation was identified in the pmrB gene (P175S) in one strain and in the parS gene (V216A) in another strain. These mutations have not been described, but they were predicted to impact the protein, and both genes are associated with colistin resistance [37]. No mutation was found in the *pmr*A, phoP, phoQ, parR, colR, colS, cprR, or cprS genes. For the 6 remaining *P. aeruginosa* strains, no mutation was found in the 10 studied genes.

### Exposure to Antibiotics Within 3 Months

Antibiotic exposure is represented as the total number of days over the 3-month follow-up period (Table 3). Overall, patients receiving inhaled colistin had greater exposure to antibiotics than patients without colistin, with 11/13 antibiotics more used in patients receiving IC.

**TABLE 3:** Exposure to antibiotics within 3 months in patients with or without inhaled colistin.

Antibiotic exposure (days)	Patients with inhaled colistin (n = 27)	Patients without inhaled colistin (n = 43)	*p*-value
Amoxicillin	119	45	<0.001
Amoxicillin-clavulanate	44	109	0.01
Cefazolin	148	59	<0.001
Ceftazidime	166	50	<0.001
Cefotaxime	35	43	0.25
Cefepim	132	128	<0.001
Piperacillin-tazobactam	3	35	<0.001
Ceftolozane-tazobactam	28	0	<0.001
Carbapenem	48	65	0.39
Levofloxacin/Ciprofloxacin	124	75	<0.001
Trimethoprim-sulfamethoxazole	59	28	<0.001
Aminoglycosides	12	2	<0.001
Linezolid	82	31	<0.001

## Discussion

Our study suggests that the use of IC as secondary prophylaxis to prevent recurrence of early *P. aeruginosa* or ESBL-PE pneumonia after LT did not promote the emergence of colistin-resistant GNB in respiratory samples via natural or acquired resistance. This report is the first prospective study to assess the risk of emergence of colistin resistance after LT. Our results are consistent with studies in non-transplanted patients, which did not observe an increased risk of colistin resistance acquisition [38–41].

Only one study evaluated the impact of IC on bronchial colonization with difficult-to-treat GNB in 70 cystic fibrosis transplant patients [42]. The authors showed that among the 15 patients who were not colonized by difficult-to-treat GNB in the immediate postoperative period, 3 of the 9 patients treated with IC prophylaxis did not develop colonization at 12 months. However, the 6 other patients who did not receive IC prophylaxis were all secondarily colonized with one or more difficult-to-treat GNB. IC did not eradicate this colonization in patients already colonized by difficult-to-treat GNB after LT. Acquired resistance to colistin was identified in only 2 of 33 patients colonized by *P. aeruginosa* and in 2 of 4 patients colonized by *Achromobacter* sp.

The lack of colistin resistance emergence when colistin is used by inhalation may be explained by the high concentrations of the antibiotic in the lung, which surpasses the MIC and the mutant prevention concentration (MPC) [43]. The MPC 90 for colistin is between 64 and 128 mg/L [44, 45]. Colistin concentrations obtained in BAL after IV administration are often below the limit of quantification, but they reach 150–180 mg/L in animals via inhalation [46, 47]. Boisson et al. showed that colistin concentrations in BAL were 100–1,000 times higher after inhalation than by IV in humans and ranged from 9.53 to 1,137 mg/L [48]. Yapa et al. also showed a higher concentration of colistin in the sputum of cystic fibrosis patients when colistin was administered via nebulization compared to the IV route [49].

We intended to determine the resistance mechanisms of colistin-resistant strains. Notably, none of the 11 sequenced strains with acquired resistance to colistin were *mcr*-positive. This result is consistent with the low prevalence of *mcr*-positive strains in France. Terveer et al. found that only two of 576 patients attending a tertiary care hospital (0.35%) were positive for *mcr*-1 in faecal samples [50]. We may have missed mcr-positive strains because of the use of a selective screening medium and the well-known existence of some colistin-susceptible mcr-positive strains [51]. We only found a mutation associated with colistin resistance in 4/11 sequenced strains (*E. coli*, K. aerogenes and 2/8 *P. aeruginosa*). Three of the 4 missense mutations found in our study concerned the two-component system PmrAB, which is largely responsible for colistin resistance via LPS modifications by the addition of cationic groups to the LPS membrane [37]. The last mutation found also concerned a two-component system, ParRS, which is also responsible for colistin resistance in *P. aeruginosa* [52]. The mechanisms of colistin resistance are primarily achieved by modification of lipid A of LPS and are not fully understood. The unexplained colistin resistance in our 7 strains may be related to mutations in other genes implicated in LPS biosynthesis [53–55] or the overexpression of efflux pumps. Some studies showed that efflux pumps contributed to colistin resistance in *E. cloacae* [56, 57], *Klebsiella pneumoniae* [58] or *Acinetobacter baumannii* [59]. To strengthen this hypothesis, Ni et al. showed that an efflux pump inhibitor, cyanide 3-chlorophenylhydrazone, suppressed and reversed colistin resistance in GNB [60].

Finally, patients receiving IC had higher morbidity during their ICU stay than patients not receiving IC. This is explained by the fact that patients were treated with IC as secondary prophylaxis after the onset of *P. aeruginosa* and ESBL-PE pneumonia in the ICU. In a retrospective before-and-after cohort analysis, we showed that patients with these pneumonias had higher morbidity in the ICU [4].

This study has several limitations. The main limitation is that it was a single-centre study with a limited number of patients. Our results primarily concerned transplant patients for COPD or ILD and cannot be generalised to patients with cystic fibrosis. The latter group are often treated with multiple lines of prolonged antibiotic therapy before LT, including colistin aerosols, for chronic colonization with *P. aeruginosa* or naturally colistin-resistant bacteria, such as *Burkholderia cepacia* [61]. The limited number of patients included in the study was compensated for by the prospective analysis of more than 800 respiratory samples. However, these samples were collected as part of the routine care for suspected pneumonia. There was no systematic respiratory sampling protocol to detect tracheobronchial colonization, thus we may have missed acquisition of resistance in asymptomatic patients. The 90-day follow-up time from LT to monitor the emergence of colistin resistance is also limited and may be evaluated more remotely in LT patients on long-term IC therapy.

## Conclusion

Our study did not find an association between the use of IC as secondary prophylaxis to prevent recurrence of early *P. aeruginosa* or ESBL-PE pneumonia after LT and an increased risk of colistin resistance in the respiratory tract. However, the efficacy of secondary prophylaxis with IC should be evaluated in a specific study to confirm the value of its use policy.

## Data Availability

The raw data supporting the conclusions of this article will be made available by the authors, without undue reservation.

## Footnotes

1
www.eucast.org

## Abbreviations

BA, bronchial aspirate; BAL, bronchoalveolar lavage; BMI, body mass index; COPD, chronic obstructive pulmonary disease; ECMO, extracorporeal membrane oxygenation; ESBL-PE, extended-spectrum β-lactamase-producing *Enterobacterales*; GNB, Gram-negative bacilli; IC, inhaled colistin; ICU, intensive care unit; ILD, interstitial lung diseases; LT, lung transplantation; MIC, minimum inhibitory concentration; MIU, million international units; MPC, mutant prevention concentration; PTC, plugged telescoping catheter; ST, sequence type.
