# Supplementary material for: No Emergence of Colistin Resistance in the Respiratory Tract of Lung Transplant Patients Treated With Inhaled Colistin
Source: Transpl Int. 2025 Jan 23;37:13545. doi: 10.3389/ti.2024.13545 (PMC11799951; doi:10.3389/ti.2024.13545)
Supplement: Supplementary file 1 [file Table1.docx]

**Table S1: Characteristics of sequenced strains with acquired colistin resistance**

NS: Not studied because not described in the species. ST: sequence type

*: Strains isolated from the same patient

Resistance genes to : Green: beta-lactams, light blue: aminoglycosides, orange: sulfonamide, purple: trimethoprim, black: macrolide, lincosamide and streptogramin B (MLS), red: quinolones, dark blue: multidrug efflux pump, brown: fosfomycin, grey: tetracycline, pink: phenicolates

| Ref. | Species | ST | Mutation in proteins implicated in colistine resistance | | | | | | | | | | | Antibiotic resistance genes |
| --- | --- | --- | --- | --- | --- | --- | --- | --- | --- | --- | --- | --- | --- | --- |
|  |  |  | PmrA | PmrB | PhoP | PhoQ | MgrB | ParR | ParS | ColR | ColS | CprR | CprS |  |
| E50 | *Escherichia coli* | ST117 / ST48 (Phylogroup F) | G53R | - | - | - | - | NS | NS | NS | NS | NS | NS | *bla*_TEM-2_, *aad*A1, *sul*2, *dfr*A1, *mdf*(A) |
| E57 | *Klebsiella aerogenes** | ST115 | G53C | - | - | - | - | NS | NS | NS | NS | NS | NS | *mdf*(A), *oqx*A, *oqx*B, *fos*A5 |
| E14 | *Enterobacter cloacae* | ST90 | - | - | - | - | - | NS | NS | NS | NS | NS | NS | *bla*_CTX-M-15_, *bla*_OXA-1_, *bla*_TEM-2_, *bla*_ACT-15_, *aph*(6)-Id, *aad*A1, *aac*(6')-Ib, *sul*2, *dfr*A14, *mdf*(A), *qnr*B1, *fos*A, *tet*(A), *cat*A1 |
| P6 | *Pseudomonas aeruginosa* | ST244 | - | - | - | - | NS | - | - | - | - | - | - | *bla*_OXA-494_, *bla*_PDC-1_, *aph*(3')-Iib, *fos*A, *cat*B7 |
| P14 | *Pseudomonas aeruginosa* | ST446 | - | - | - | - | NS | - | - | - | - | - | - | *bla*_OXA-395_, *bla*_PDC-16_, *aph*(3')-Iib, *fos*A, *cat*B7 |
| P24 | *Pseudomonas aeruginosa* | ST253 | - | - | - | - | NS | - | - | - | - | - | - | *bla_OXA-488_, bla*_PDC-34_, *aph*(3')-Iib, *fos*A |
| P27 | *Pseudomonas aeruginosa* | ST2222 | - | - | - | - | NS | - | - | - | - | - | - | *bla*_OXA-494_, *bla*_PDC-45_, *aph*(3')-Iib, *fos*A, *cat*B7 |
| P56 | *Pseudomonas aeruginosa* | ST244 | - | P175S | - | - | NS | - | - | - | - | - | - | *bla_OXA-494_, bla*_PDC-1_, *aph*(3'')-Ib, *aph*(3')-Iib, *fos*A, *cat*B7 |
| P59 | *Pseudomonas aeruginosa** | ST new | - | - | - | - | NS | - | - | - | - | - | - | *bla_OXA-395_, bla*_PDC-19a_, *aph*(3')-Iib, *fos*A, *cat*B7 |
| P77 | *Pseudomonas aeruginosa* | ST395 | - | - | - | - | NS | - | V216A | - | - | - | - | *bla_OXA-395_, bla*_PDC-8_, *aph*(3')-Iib, *sul*1, *fos*A, *cat*B7 |
| P79 | *Pseudomonas aeruginosa* | ST319 | - | - | - | - | NS | - | - | - | - | - | - | *bla*_OXA-488_, *bla*_PDC-16_, *aph*(3')-Iib, *fos*A, *cat*B7 |
